# Impact of Video Modules-Based Training on Knowledge, Attitude, and Practices of Cleaning and Disinfection Among Housekeeping Staff at a Tertiary Care Center During the COVID-19 Pandemic

**DOI:** 10.7759/cureus.19125

**Published:** 2021-10-29

**Authors:** Vanya Singh, Himanshu Narula, Sakshi Supehia, Maneesh Sharma, Puneet K Gupta, Anita Sharma, Shalinee Rao

**Affiliations:** 1 Microbiology, All India Institute of Medical Sciences, Rishikesh, IND; 2 Microbiology, Rifleman Jaswant Singh Rawat (RJSH) COVID Hospital, All India Institute of Medical Sciences, Rishikesh, IND; 3 Public Health, All India Institute of Medical Sciences, Rishikesh, IND; 4 College of Nursing, All India Institute of Medical Sciences, Rishikesh, IND; 5 Microbiology, All India Institute of Medical Sciences, Bilaspur, IND; 6 Hospital Infection Control, All India Institute of Medical Sciences, Rishikesh, IND; 7 Pathology, All India Institute of Medical Sciences, Rishikesh, IND

**Keywords:** knowledge-attitude-practice theory, covid-19 infection control, hospital infection control practice, kap, healthcare workers, disinfection, cleaning, housekeeping staff, covid-19

## Abstract

Aim

To assess the knowledge, attitude, and practice toward cleaning and disinfection among housekeeping (HK) staff amid the coronavirus disease 2019 (COVID-19) pandemic.

Methods

A quasi-experimental was undertaken among HK staff at a tertiary care hospital. A 30-item structured questionnaire was used in the study, which consisted of questions pertaining to knowledge (11), attitude (8), and practice (11) toward cleaning and disinfection.

Result

One-hundred-two participants were included by convenient sampling, with mean age = 30-37 years. There was an improvement in pre-test and post-test knowledge (6.21 to 9.7) and practice score (9.97 to post-test 10.52). However, the attitude score did not show a significant change in the post-test score (p=0.964), showing that they were having a positive attitude toward the practices before training too.

Conclusion

Improvement in the post-test score shows that periodic targeted training sessions on cleaning and disinfection among housekeeping staff help improve their knowledge, attitude, and practices toward infection prevention and control (IPC) during the COVID-19 pandemic, thus minimizing the spread of the virus in a hospital environment, reducing their apprehension, and preparing them to work in such pandemic situations.

## Introduction

Severe acute respiratory syndrome coronavirus 2 (SARS-CoV-2) is the causative agent of the transmissible coronavirus disease 2019 (COVID-19), which was first detected in Wuhan, China, in 2019 and was declared a pandemic by the World Health Organization (WHO) on March 11, 2020 [1]. SARS-CoV-2 is transmitted mainly through close contact and respiratory droplets, whereas airborne transmission may be possible during aerosol-generating medical procedures [2-3]. Environmental surfaces act as potential reservoirs for the transmission of COVID-19 [2-4]. Many studies showed that viable virus particles are potentially infectious, can be detected on surfaces, but the extent to which people can be infected by exposure to contaminated surfaces remains to be determined [5].

Studies have also demonstrated that the SARS CoV-2 virus can remain viable on surfaces for three to five days and is susceptible to standard disinfection methods, including household bleach, at normally used concentrations. Solutions containing at least 70% alcohol can inactivate SARS-CoV-2 in less than five minutes [6-7].

Various studies suggest that environmental contamination by SARS-CoV-2 in the hospital around COVID-19 patients is extensive [6-7] and the hospital environment could potentially be a source of virus spread, among health care workers (HCWs), patients, and visitors [8-9].

In order to prevent the transmission of healthcare-associated infections (HAIs), including SARS-CoV-2, hospital infection prevention and control (IPC) procedures should be strictly followed.

Housekeeping (HK) workers, though often undervalued and overlooked, are the backbone of routine cleaning and disinfection in any healthcare facility, thus having a major role in the prevention of HAIs. Studies suggest that interventions directed at environmental service workers result in significant improvement in cleaning and disinfection [10-11]. In the midst of COVID-19, housekeeping staff act as frontline workers with the challenging responsibilities of vigorous cleaning of high-touch surfaces in healthcare facilities despite having apprehension of working in COVID-19 areas. For optimal effectiveness of environmental cleaning and disinfection measures, HK workers need to be aware of environmental disinfection and be willing to learn and improve their practices. This is in accordance with the “Knowledge-Attitude-Practice (KAP) theory,” which states that changes in human practices are a step-wise process that involves the acquisition of knowledge, the subsequent generation of positive attitudes, and, finally, the adoption of practice [12]. Understanding the knowledge, attitudes, and practice of HK staff regarding cleaning and disinfection practices is important to tailor the training of HK workers according to their specific needs and allay their apprehension while working in COVID-19 areas.

In the present study, we conducted video module-based training sessions of the HK workers department working in different areas of the hospital. Their knowledge, attitudes, and practice regarding environmental cleaning and disinfection were assessed by taking a pre-test and post-test during each session. This was important in the context of this KAP study, as housekeeping staff are the backbone of the hospital infection control system. It was equally important to know whether they were able to perform their respective duties efficiently during the COVID-19 pandemic without any undue apprehension. To the best of our knowledge, there are very limited studies on this particular subgroup in a tertiary care center in India. 

## Materials and methods

Methodology

A quasi-experimental study with a single-group pre-test and post-test design was conducted among HK staff with the aim to assess the knowledge, attitude, and practice regarding cleaning and disinfection during the current COVID-19 pandemic in a tertiary care institute for a period of one week, from 21 April to 28 April 2020. The training consisted of three events: pre-test, video-assisted training, and post-test.

The study was conducted among HK staff after approval from the institutional ethics committee vide letter no. AIIMS/IEC/20/162, dated 11/04/2020, and after obtaining written informed consent of the participants. The confidentiality of information and anonymity of the participants was maintained. Study participants were included by convenience sampling and a total of 102 HK staff posted in different COVID-19 areas and willing to participate were recruited as study participants. Those who were absent at the time of data collection and intervention were excluded from the study.

Study tool

A semi-structured questionnaire with four sections was used to collect the data; the first section was the demographic information of participants followed by knowledge, attitude, and practice questions regarding the cleaning and disinfection of the hospital. The questionnaire was prepared by experts in the field of microbiology and nursing of the hospital infection control committee, based on the cleaning and disinfection policy of the institute. Although the content of training and questionnaire was revised thoroughly by the Hospital Infection Control team members, content validation could not be done due to a shortage of time. The institutes' cleaning and disinfection policy draft was formulated based on guidelines of the World Health Organization (WHO), Center for Disease Control (CDC), Ministry of Health and Family Welfare Government of India (MoHFW, GOI), and ICMR, scientific literature, and available logistics of the institute [13-17].

A pre-test questionnaire was conducted for 20 minutes before the training session. The training session included videos regarding cleaning and disinfection practices to be followed at the hospital along with interactive sessions in the local language (Hindi). Later, the videos were made available on all desktops of the institute’s hospital wards as well as on the YouTube channel with its link on the institute’s website [18-20]. The post-test session was concluded with the distribution of the post-test questionnaire, which was collected after 30 minutes (Appendices 1-2).

The participants were trained in small groups of 25-30 at a time to avoid mass gatherings. All were supposed to follow the COVID protocol, with a compulsory wearing of masks at all times. A total of four such training sessions were taken. A few of the HK staff were not literate enough to read and attempt the questionnaires. For such participants, a few HCWs were requested to help them fill their tests by interviewing them.

The KAP questionnaires consisted of 11 questions of multiple-choice type pertaining to knowledge, eight questions catering to attitude on a Likert scale of 1-4, and 11 questions concerning practices toward cleaning and disinfection in the hospital amid the COVID-19 pandemic with ‘Yes’ and ‘No’ responses. An arbitrary tool was devised to assess the knowledge, attitude, and practices of HK staff regarding cleaning and disinfection. Knowledge was assessed by giving a score of one to each correct answer and 0 to the wrong answer. The knowledge score ranged from a maximum of 11 scores to a minimum of 0. Scores < 7 were taken as “poor” and ≥7 as “adequate” knowledge of cleaning and disinfection among housekeeping staff. Similarly, the attitude was assessed by giving a score of 1 to 5 for the responses of strongly disagree to strongly agree. Scores <5 were taken as “negative” and ≥5 as “positive.” Likewise, for practice, a score of 1 was given to the response of Yes, depicting adequate/good practice, and 0 to the response of No, depicting poor practice. Scores < 7 were taken as “poor” and ≥7 as “adequate” practices of cleaning and disinfection among housekeeping staff.

Data analysis

Data were collected and entered in Microsoft Excel (Microsoft Corporation, Redmond, WA), and missing entries were excluded. Statistical analysis was done to assess the pre-test and post-test scores using SPSS (IBM SPSS Statistics for Windows, Version 21.0; IBM Corp., Armonk, NY). Descriptive analysis was done for frequencies and percentages. Data were checked for normality using the Shapiro Wilk test, which concluded that the data were not normally distributed. Therefore, the Wilcoxon-signed rank test was used to evaluate the mean of the pre and post-test scores of housekeeping staff. P<0.05 was considered statistically significant.

## Results

A total of 102 housekeeping staff participated in this one-group pre and post-test quasi-experimental study. The mean age of participants was 30.48 ± 6.58 years. Among the participants, 53 (52%) were males, 48 (47%) had primary education, 83 (81.4%) had previous training, and 67 (65.7%) had experience of one to five years. The detailed demographic characteristics of the participants are shown in Table 1.

**Table 1:** Demographic characteristics of the study participants (N=102)

Demographic variables	Categories	Frequencies	% age
Age	< 25 Year	28	27.5
	25 - 35 Year	40	39.2
	> 35 Year	34	33.3
Sex	Male	53	52.0
	Female	49	48.0
Education	Primary	48	47.1
	Secondary	39	38.2
	Sr. Secondary	11	10.8
	Graduate	4	3.9
Area of Work	COVID IPD	22	21.6
	Screening OPD	4	3.9
	Laboratory	5	4.9
	Others	71	69.6
Previous Training	Yes	83	81.4
	No	19	18.6
Years of Experience	< 1 Year	17	16.7
	1-5 Years	67	65.7
	> 5 Years	18	17.6

Assessment of knowledge

**Figure 1:**
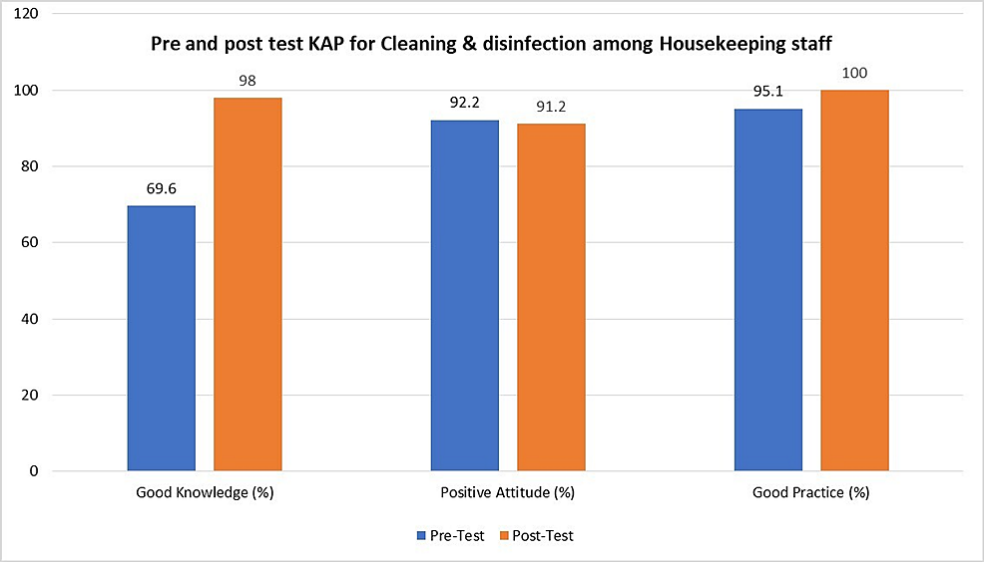
Pre and post-test KAP of housekeeping staff regarding cleaning and disinfection KAP: Knowledge-Attitude-Practice

A total of 11 questions were used to measure the knowledge of cleaning and disinfection among housekeeping (Table 1). The pre-test mean knowledge score of participants was 6.21 (SD= 2.09), which increased to 9.71 (1.5) in the post-test after the intervention. The increase in knowledge mean score was statistically significant with a p-value = 0.001 (Tables 2-3). The pre-test and post-test scores are demonstrated in Table 4. Prior to the training session, the level of knowledge was good in only 69.6% of participants, which improved impressively to 98% post-training (Figure 1).

**Table 2:** Comparison of mean pre-test and post-test scores for knowledge, attitude, and practice toward cleaning and disinfection of housekeeping staff # Wilcoxon signed ranks test done

		Mean	S.D	Paired T-test	P-value
Knowledge	Pre-test	6.21	2.09	13.15	0.001*
	Post-test	9.71	1.5		
Attitude	Pre-test	7.00	1.58	0.39	0.964
	Post-test	6.99	1.88		
Practice	Pre-test	9.97	1.95	3.099	0.003*
	Post-test	10.52	0.87		

**Table 3:** Comparison of scores before and after training using the Wilcoxon signed-rank test a. Post-test score > Pre-test score, b. Post-test score < Pre-test score, c. Post-test score = Pre-test score

	Ranks		Ties	p-value
	Positive	Negative		
Post-Test Knowledge Score – Pre-Test Knowledge Score	89^a^	4^b^	9^c^	0.001*
Post-Test Attitude Score – Pre-Test Attitude Score	34^a^	28^b^	40^c^	0.389
Post-Test Practice Score – Pre-Test Practice Score	30^a^	14^b^	58^c^	0.003*

**Table 4:** Test scores for knowledge before and after training # McNemar Test, * p-value < 0.05 Note: Knowledge was assessed by assigning 1 to the correct answer and 0 to the wrong answer. The scale measured knowledge from maximum 11 to minimum 0. Scores < 7 were taken as poor and ≥ 7 as adequate knowledge of cleaning and disinfection among housekeeping staff.

	Questions	Pre- Test		Post- Test		
S. No.	Knowledge	Correct N (%)	Incorrect N (%)	Correct N (%)	Incorrect N (%)	p- value^#^
1	Prerequisite before entering the COVID-19 area is to wear	79 (77.5)	23 (22.5)	101 (99)	1 (1)	.001*
2	Recommended frequency of mopping the floor and frequently touched surfaces in COVID-19 area?	64 (62.7)	38 (37.3)	98 (96.1)	4 (3.9)	.001*
3	What is the correct method of mopping in a COVID area?	85 (83.3)	17 (16.7)	101 (99)	1 (1)	.001*
4	What should be done to stop people before starting cleaning in any COVID area?	92 (90.2)	10 (9.8)	101 (99)	1 (1)	.012*
5	The correct sequence of cleaning the mop using the triple bucket technique is:	37 (36.3)	65 (63.7)	94 (92.2)	8 (7.8)	.001*
6	What is the correct concentration of phenyl solution in the 3^rd^ bucket for cleaning?	50 (49)	52 (51 %)	86 (84.3)	16 (15.7)	.001*
7	0.5% sodium hypochlorite solution is recommended for cleaning & disinfection. Which composition is equivalent to recommended composition?	68 (66.7)	34 (33.3)	86 (84.3)	16 (15.7)	.001*
8	Which disinfectant solution is to be used to clean highly touched metallic surfaces like doorknobs in hospitals?	38 (37.3)	64 (62.7)	82 (80.4)	20 (19.6)	.001*
9	What will be the frequency to clean highly touched metallic surfaces, like doorknobs, in hospitals?	48 (47.1)	54 (52.9)	91 (89.2)	11 (10.8)	.001*
10	Which of the disinfectant solutions will be used to clean less frequently touched surfaces, like tables/chairs, in hospitals?	27 (26.5)	75 (73.5)	67 (65.7)	35 (34.3)	.001*
11	What will be the frequency to clean less frequently touched surfaces in hospitals?	45 (44.1)	57 55.9)	83 (81.4)	19 (18.6)	.001*

Assessment of attitude

For the assessment of attitude, the participants (n=102) were asked eight questions. Before training, the mean score of attitudes in the pre-test was 7± 1.58 with no significant change post-intervention (Tables 2-3). About 95% of participants were having a positive attitude regarding the use of phenyl and sodium hypochlorite solution for cleaning and disinfection for infection containment and performing all practices as per institute guidelines and protocols. Prior to training, 31.4% had a negative attitude toward cleaning products used for disinfection and believed that the disinfectants might be harmful to them. This negative attitude improved slightly after training (Table 5). After conducting training, the mean score of attitudes in post-test was 6.99 ± 1.88, which was not statistically significant (P = 0.964, Tables 2-3).

**Table 5:** Test scores for practice before and after training # McNemar Test, * p-value < 0.05 Note: Practice was assessed by giving 1 to the response of Yes, depicting adequate practice, and 0 to a response of No, depicting poor practices. Scores <7 were taken as poor, ≥7 as adequate practices of cleaning and disinfection among housekeeping staff.

	Questions	Pre- Test		Post- Test		
S. No.	Practice	Adequate N (%)	Poor N (%)	Adequate N (%)	Poor N (%)	p- value^#^
1	Do you wear all the necessary PPEs for cleaning and disinfection in the hospital?	85 (83.3)	17 (16.7)	92 (90.2)	10 (9.8)	0.143
2	Are you using the triple-bucket technique for cleaning in hospitals in your daily routine?	87 (85.3)	15 (14.7)	92 (90.2)	10 (9.8)	0.359
3	Do you place barrier signage before starting to mop?	90 (88.2)	12 (11.8)	99 (97.1)	3 (2.9)	0.022*
4	Do you sweep the dust first before starting mopping?	95 (93.1)	7 (6.9)	100 (98)	2 (2)	0.125
5	Are you discarding the liquid waste from all the buckets after cleaning into effluent treatment?	98 (96.1)	4 (3.9)	97 (95.1)	5 (4.9)	1.000
6	Are you cleaning the outer surface of each bucket in the triple bucket system with 0.5% sodium hypochlorite after cleaning the floor with phenyl solution?	92 (90.2)	10 (9.8)	100 (98)	2 (2)	0.021*
7	Do you clean floor surfaces in the COVID-19 area every 4 hourly?	97 (95.1)	5 (4.9)	101 (99)	1 (1)	0.219
8	Are you cleaning high-touch surfaces like doorknobs every 4 hours?	96 (94.1)	6 (5.9)	94 (92.2)	8 (7.8)	0.774
9	Are you cleaning the low-touch surfaces like tables and chairs every week?	96 (94.1)	6 (5.9)	99 (97.1)	3 (2.9)	0.453
10	Are you changing the disinfectant solution and mop after cleaning every 240 square feet floor area?	93 (91.2)	9 (8.8)	101 (99)	1 (1)	0.008*
11	Are you dipping the used mop in 0.5% sodium hypochlorite solution for 30 min and drying it before?	88 (86.3)	14 (13.7)	98 (96.1)	4 (3.9)	0.013*

Assessment of practices

Practices regarding cleaning and disinfection among housekeeping staff were assessed by 11 questions. The mean score of practices was 9.97 in pre-test and 10.52 in post-test. The mean difference was statistically significant with a p-value of 0.003 (Table 2, Table 6). More than 95% of participants affirmed adequate practice regarding all questions post-training, except regarding wearing of necessary PPE during cleaning (9.8% poor practice), cleaning using the triple-bucket system (9.8% poor practice), and cleaning of high-touch surfaces every four hourly (7.8% poor practice) (Table 3).

**Table 6:** Test scores for attitude before and after training # McNemar test Note: Attitude was assessed by giving 1 to the responses strongly agree and agree, depicting a positive attitude, and 0 to a response of strongly disagree and disagree, depicting a negative attitude. Scores < 5 were taken as negative, ≥ 5 as a positive attitude toward cleaning and disinfection among housekeeping staff.

	Questions	Pre-Test		Post-Test		
S. No.		Positive Attitude N (%)	Negative Attitude N (%)	Positive Attitude N	Negative Attitude N (%)	p- value^#^
1	Cleaning and disinfection work is very important for the safety of healthcare staff.	95 (93.1)	7 (6.9)	91 (89.2)	11 (10.8)	0.424
2	Are the cleaning products used for disinfection by housekeeping staff may be harmful to them?	70 (68.6)	32 (31.4)	75 (73.5)	27 (26.5)	0.52
3	Wearing PPE while cleaning and disinfecting has proven beneficial for the housekeeping staff.	94 (92.2)	8 (7.8)	94 (92.2)	8 ( 7.8)	1.000
4	The triple bucket technique can be used routinely for cleaning and disinfection in hospitals.	91 (89.2)	11 (10.8)	91 (89.2)	11 (10.8)	1.000
5	Using phenyl and sodium hypochlorite solution while cleaning and disinfection is essential for infection containment?	97 (95.1)	5 (4.9)	96 (94.1)	6 (5.9)	1.000
6	Is it right to disinfect floors and highly touched surfaces in COVID-19 areas every four hours?	93 (91.2)	9 (8.8)	94 (92.2)	8 (7.8)	1.000
7	Performing cleaning and disinfection practices as per the institute’s protocol will prevent infection spread.	97 (95.1)	5 (4.9)	93 (91.2)	9 (8.8)	0.388
8	All the liquid waste after cleaning should be discarded in the effluent treatment system of the hospital.	77 (75.5)	25 (24.5)	79 (77.5)	23 (22.5)	0.85

On applying the Wilcoxon signed-rank test, it was observed that the difference in pre and post-test scores was statistically significant.

## Discussion

To the best of our knowledge, this is the first study especially targeting the HK staff working in healthcare facilities during the current COVID-19 pandemic. The surge of COVID-19 cases has emphasized the importance of environmental cleaning in hospitals and thus the role of HK workers as pillars for primordial prevention. Formal training in infection control is conducted by some hospitals as a component of preservice compulsory training, however, regular reinforcement training to provide skill-based training for empowering the HK workers is usually not considered.

In our study, 47% had either primary level education or less while about 38.2% of HK workers had at least secondary level education. Ni K et al. showed, that in their study, 60.5% of sanitation workers were uneducated or with primary education [21]. We did not find a correlation between workers’ age, gender, years of experience, preservice training, or education level, and their knowledge in our study suggests that the observed pre/post changes were due to the training intervention and not due to differences in participant characteristics. A previous study by Boyce et al. demonstrated that continuous education and feedback improved disinfection by our housekeeping staff [22].

Previous studies also suggest that assessment and feedback of cleaning performance is a critical part of environmental infection prevention [23-24]. Our study findings are of reinforcement training focusing on cleaning procedures and 81.4% of the workers had received training previously during their service similar to the study findings of Ni et al. [21]. Although most of the participants (81.4%) affirmed that they participate in one or more training related to infection prevention and control practices, some (18.6%) started their activities without any prior training. These results indicate that the hospital HK workers may serve as a potential risk for transmission of COVID-19 as well as other healthcare-associated infections.

The main aim of conducting reinforcement training in this study was to educate HK workers and improve their knowledge and practices. Previous studies have also found that educational interventions alone may not lead to sustained improvements in hospital cleaning and disinfection and that sustained optimal improvement may be achieved only when education is ongoing and additional strategies are implemented [11,25-26]. The results of our study suggest that training intervention met the need of the hour and significant improvement in baseline knowledge was noted. Significant improvement in the knowledge of HK workers with respect to aspects of the use of personal protective equipment, the frequency for cleaning of high-touch surfaces in COVID areas, and the correct technique of mopping was also noted. For several questions, more than 90% of participants answered correctly. In our study, 83.3% of the HK workers were aware of necessary PPE prior to training. Similar post-intervention observations have been reported by Martin et al. [27]. There is often a lack of awareness amongst cleaning staff, regarding occupational safety and health, including the use of PPE such as mask and rubber or latex gloves, and reluctance to use them as they felt that using PPE affected the efficiency of their cleaning practices [21]. However, in our study, participants were found to be willing to follow correct practices after receiving training. There was no significant change in the attitude of the staff post-intervention, which can be explained by a high baseline positive attitude in the pre-training phase.

In this study, training given to HCWs was assisted with various video modules and was found to be very effective and practical. Similar video-assisted training has been found to be useful for training HCWs in various studies [28-29]. We were also able to identify the gaps in our training program by analyzing the pre and post-training assessments. Although significant gain in knowledge was appreciated in all questions, there was still scope of improvement regarding a few aspects like the concentration of various disinfectants to be used and cleaning of low-touch surfaces and metallic surfaces.

Targeted surface disinfection with a special focus on frequently touched surfaces is indispensable in the current scenario. The role of daily disinfection of high-touch surfaces in isolation rooms as an adjunctive measure to reduce transmission has previously been suggested by Kundrapu et al. [30]. However, disinfectants may be hazardous to HCWs and patients, as well as to the environment, and require special safety precautions. The correct use of disinfectant products is crucial for the control of HAIs so information on the products commonly used in healthcare facilities should be easily available. The cleaning staff must essentially be well-versed with information regarding when, where, and how different products need to be applied. In our study, participants had poor baseline knowledge regarding disinfectants, their use, and concentration. This could be due to poor primary-level education among HKs. Although statistically significant improvement was observed post-intervention, only 65.7% of participants were aware of the disinfection of low-touch surfaces and 80.4% knew about the disinfection of metallic surfaces, highlighting the scope for improvement and need for reinforcement in this domain.

Most of the literature related to the optimum environmental cleaning in healthcare systems and knowledge and practices of cleaning workers comes from countries with relatively plentiful resources. In resource-limited healthcare settings, additional challenges exist, such as inadequate infrastructure, logistics, or limited or uneducated staff, etc., which contribute to inadequate cleaning of environmental surfaces in healthcare settings [21,26]. Studies examining the level of knowledge and application of adequate IPC practices by HK workers during the current COVID-19 pandemic are largely absent in the literature, with comparisons not possible. In view of several knowledge gaps and high-risk cleaning and disinfection practices found amongst HK staff at our institute, we recommend that similar studies should be done to identify gaps in knowledge about safe and effective practices in other resource-constrained healthcare settings. The paucity of resources at our institute, a central government center, during the ongoing COVID pandemic may be reflective of the situation across smaller districts or municipality hospitals in India. Tailored prevention messages should highlight identified gaps and provide targeted information using digital or social media regarding safe cleaning and disinfection.

The strength of our study is the intervention in form of the in-house video in the local language (Hindi) designed specifically for frontline HK workers, addressing common barriers to the implementation of best practices, which was well-received by the participants. The limitation of our study was that being a single-center study with a small sample size, our findings may not be generalized. Another limitation of this study was that the extent of the perceived risk of contracting COVID-19 among the HCWs could not be assessed, which is relevant for understanding the attitude and cleaning practices of HKs. Also, we could not assess the actual practices of workers by observing them during work, rather only assessed through conducting questionnaires. We recommend that covert observation may be done to assess cleaning practices of staff members for future studies. It is also important to take the feedback of the HK workers regarding their needs and training provided along with an assessment of available logistics.

## Conclusions

This article presents an understanding of cleaning and disinfection practices in HK staff members. In this underexplored group of healthcare workers, the results of our study suggest that they had a high level of knowledge regarding cleaning and disinfection, with a good attitude toward working in COVID-19 areas, and were willing to follow good practices if provided with necessary cleaning equipment and protective gear. They also understand the importance of their role in the healthcare facility, which is indeed critical to prevent the transmission of infections in a healthcare facility.

## Appendices

Appendix 1: Youtube Link of video modules used for training

Video Content Link

1. Do’s and Don’ts, Hand Hygiene: https://www.youtube.com/watch?v=xb_YP5P05es&feature=youtu.be

2. Donning and Doffing of PPE: https://www.youtube.com/watch?v=DLuLzQ9JDoQ&feature=youtu.be

3. Cleaning and disinfection: https://www.youtube.com/watch?v=isAOyzGk8ak&feature=youtu.be

Appendix 2: Questionnaire for assessment of the knowledge, attitude, and practices of HK staff regarding cleaning and disinfection

Sociodemographic Profile Sheet of Participant

ID No:

Date:                                                                                    Time:

Undertaking:

I______________________ Son/Daughter of Shri ______________________ Age ___ years resident of _________________________________________do hereby affirm and declare that the information given above is true and correct to the best of my knowledge and belief. I have not concealed or falsified any information.Consent:

I give consent to use the above-provided information for the improvement of teaching/ training in future courses and for research purposes if required. I share this information under the investigator's declaration that my identity will not be disclosed.

Yes ( )                    No ( )

Signature with date & Name of participant

Instructions- Please read the statement carefully and tick mark (√) for the most appropriate option and fill the blanks.

1. Age (In years) ………………………..

2. Gender

a)  Male

b)  Female

c)  Other

3. Education

a)     Primary 

b)    Secondary/

c)    Sr. Secondary

d)    Graduated / Post Graduated 

4. Area of Work

a) Inpatient COVID Area

b) Screening COVID Area

c) Laboratory COVID Area

d) Others

5. Duration of Work (In Days)

a) COVID Block ……………..

b) Non-COVID Block ………

6. Years of experience as housekeeping staff (in years) 

a) At AIIMS Rishikesh …………

b) Other Hospital ………….

8. Undergone for any in-service training of cleaning & disinfection in past………….

SECTION A: STRUCTURED KNOWLEDGE QUESTIONNAIRE

Instruction for the participant's Code no:

Kindly go through each questionnaire carefully and put a tick (✓) mark against the most suitable answer. Your identity will be kept confidential.

1.    Prerequisite before entering the COVID-19 area is to wear:

a)    Mask

b)    Cap

c)    Gloves

d)    Full PPE

2.    Recommended frequency of mopping the floor and frequently touched surfaces in COVID-19 area?

a)    Every two hours

b)    Every four hours

c)    Every 24 hours

d)    Every 8 hours

3.    What is the correct method of mopping in a COVID area?

a)    Figure of 8

b)    Parallel Line

c)    Straight Line

d)    Figure of 4

4.    What should be done to stop people before starting cleaning in any COVID area?

a)    Use signage with cleaning in progress

b)    Will inform them verbally

c)    Free movement is acceptable.

5.    Correct sequence of cleaning the mop using the triple-bucket technique is:

a)    1st bucket disinfectant, 2nd water, 3rd detergent solution

b)    1st bucket water, 2nd disinfectant, 3rd detergent solution

c)    1st bucket water, 2nd detergent, 3rd disinfectant solution

d)    1st bucket detergent, 2nd water, 3rd disinfectant solution

6.    What is the correct concentration of phenyl solution in the 3rd bucket for cleaning?

a)    100 ml phenyl in 9 liters of water

b)    100 ml phenyl in 10 liters of water.

c)    500 ml phenyl in 9 liters of water.

d)    500 ml phenyl in 10 liters of water.

7.    0.5% Sodium hypochlorite solution is recommended for cleaning & disinfection. Which composition is equivalent to recommended composition?

a)    1 liter of 5% sodium hypochlorite in 9 liters of water.

b)    1 liter of 5% sodium hypochlorite in 10 liters of water.

c)    5 liters of 5% sodium hypochlorite in 9 liters of water.

d)    5 liters of 5% sodium hypochlorite in 10 liters of water.

8.    Which disinfectant solution is to be used to clean highly touched metallic surfaces like doorknobs in hospitals?

a)    0.5 % sodium hypochlorite

b)    0.1% sodium hypochlorite

c)    70 % Alcohol

d)    30% Alcohol

9.    What will be the frequency to clean highly touched metallic surfaces, like doorknobs, in the hospital?

a)    Every two hours

b)    Every four hours

c)    Every 24 hours

d)    Every 8 hours

10. Which of the disinfectant solutions will be used to clean less frequently touched surfaces,

like tables/chairs, in the hospital?

a)    0.5 % sodium hypochlorite

b)    0.1% sodium hypochlorite

c)    70 % alcohol

d)    30% alcohol

11. What will be the frequency to clean less frequently touched surfaces in the hospital?

a)    Every day

b)    Every week

c)    Every month

d)    Every year

SECTION B: STRUCTURED ATTITUDE QUESTIONNAIRE

Instruction for the participant's  code no:

Kindly go through each attitude questionnaire carefully and put a tick mark (✓) against the most suitable answer. Your identity will be kept confidential.

Q. No.

Questions on Attitude

Strongly agree/ Disagree/ Agree/ Strongly agree

1. Cleaning and disinfection work is very important for the safety of healthcare staff.

2. Are the cleaning products used for disinfection by housekeeping staff may be harmful to them?

3. Wearing PPE while cleaning and disinfection practices are proven beneficial for the housekeeping staff.

4. Triple bucket technique can be used routinely for cleaning and disinfection in hospitals.

5. Using phenyl and sodium hypochlorite solution while cleaning and disinfection are essential for infection containment?

6. Is it right to disinfect floors and highly touched surfaces in COVID-19 Area every four hours.

7. Performing cleaning and disinfection practices as per the institute’s protocol will prevent infection spread.

8. All the liquid waste after cleaning should be discarded in the effluent treatment system of the hospital.

SECTION C: PRACTICE CHECKLIST FOR CLEANING & DISINFECTION

Instruction for the participant's Code no:

Kindly go through each attitude questionnaire carefully and put a tick mark (✓) against the most suitable answer. Your identity will be kept confidential.

Practice Steps: Yes/ No

1. Do you wear all the necessary PPEs for cleaning and disinfection in the hospital?

2. Are you using the triple bucket technique for cleaning in hospitals in your daily routine?

3. Do you place barrier signage before starting to mop?

4. Do you sweep the dust first before starting mopping?

5. Are you discarding the liquid waste from all the buckets after cleaning into effluent treatment.

6. Are you cleaning the outer surface of each bucket in the triple bucket system with 0.5% sodium hypochlorite after cleaning the floor with phenyl solution?

7. Do you clean floor surfaces in the COVID-19 area every 4 hourly?

8. Are you cleaning high-touch surfaces like doorknobs every 4 hours?

9. Are you cleaning the low-touch surfaces like tables and chairs every week?

10. Are you changing the disinfectant solution and mop after cleaning every 240 square feet floor area?

11. Are you dipping the used mop in 0.5% sodium hypochlorite solution for 30 min & drying it before?
